# Data on physicochemical properties of LPEI 25 kDa, PEI“Max” 40 kDa and PEIpro™

**DOI:** 10.1016/j.dib.2016.05.062

**Published:** 2016-06-01

**Authors:** Laurence Delafosse, Ping Xu, Yves Durocher

**Affiliations:** a Département de biochimie et médecine moléculaire, Faculté de Médecine, Université de Montréal, QC, Canada; b National Research Council of Canada, Building Montreal-Royalmount, 6100 Royalmount Avenue, Montreal, QC, Canada H4P 2R2

## Abstract

The data presented in this article are related to the research article entitled “Comparative study of polyethylenimines for transient gene expression in mammalian HEK293 and CHO cells” (Delafosse et al., 2016 [1]). Polyethylenimine is a cationic polymer whose linear form has been described as the most efficient to transfect a wide range of cell lines and thus is broadly used in transient gene expression. Data presented in this article compares apparent size and polydispersity as determined by size exclusion chromatography of three commercially available linear PEIs, namely LPEI, PEI“Max” and PEIpro™. Impact of those features on plasmid DNA affinity was established by plasmid DNA agarose gel migration assay.

**Specifications Table**

**Table:** 

Subject area	*Biology*
More specific subject area	*Cationic polymer*
Type of data	*Figure*
How data was acquired	*Size exclusion chromatography (GE ÄKTA explorer, Amersham Pharmacia)*
	*Gel agarose electrophoresis*
Data format	*Analyzed*
Experimental factors	*Solutions of polyethylenimines were prepared at a concentration of 1 mg/mL*
Experimental features	*Apparent size and plasmid DNA affinity of LPEI, PEI“Max” and PEIpro™ polymers*
Data source location	*Montreal, Quebec, Canada*
Data accessibility	*The data is with this article*

## Value of the data

• This is the first report comparing the apparent size of the LPEI 25 kDa, PEI”Max” and PEIpro™ polymers.
• Data provides qualitative evidence on the degree of acylation and polydispersity of LPEI 25 kDa, PEI“Max” and PEIpro™ polymers.
• Data indicates that PEIpro™ is the most effective to condense DNA compared to LPEI and PEI“Max” at physiological pH.

## 1 Data

**Fig. 1:**
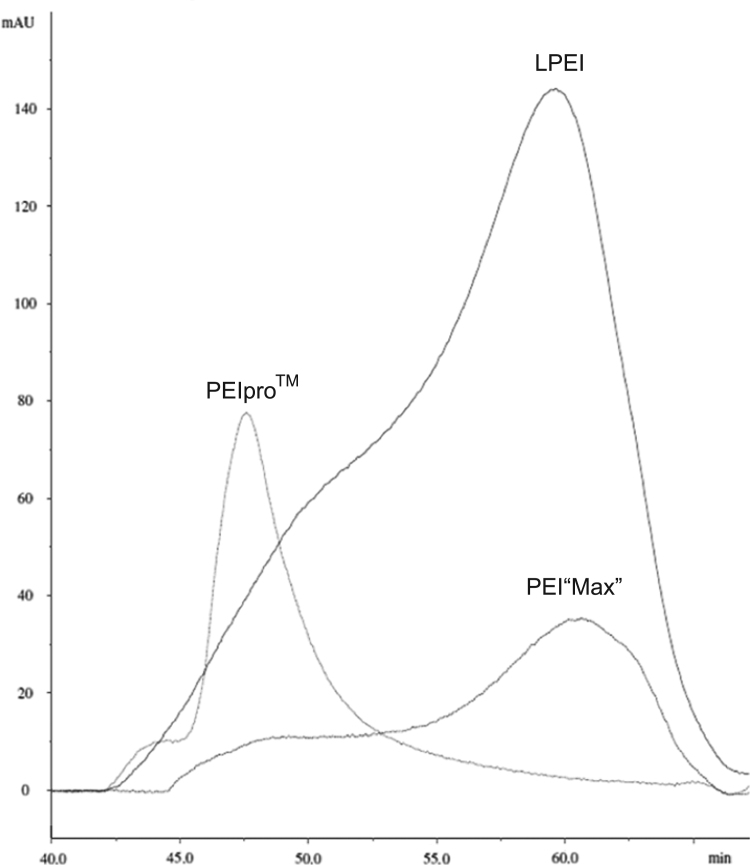
Size exclusion chromatography of LPEI 25 kDa, PEI“Max” 40 kDa (hydrochloride salt) and PEIpro™.

**Fig. 2:**
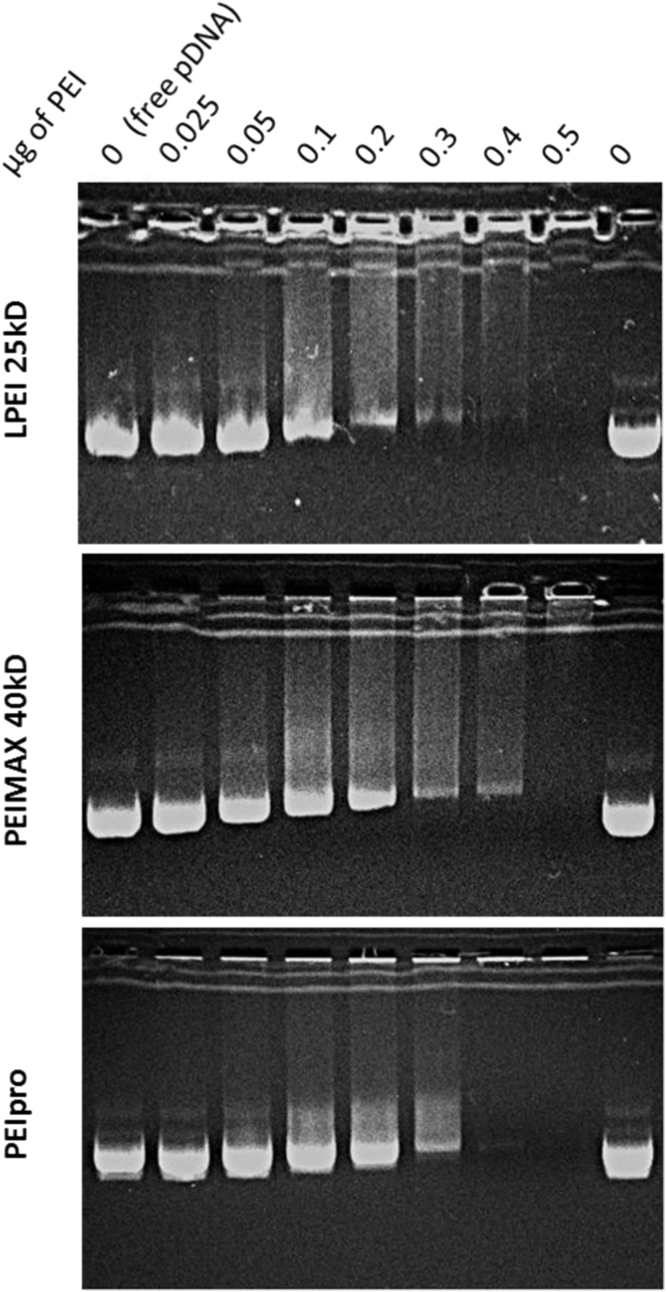
DNA condensation ability of polyethylenimines.

PEIs are widely used to introduce a gene of interest into mammalian cells [2]. The data presented in this article includes size exclusion chromatography (SEC) and DNA affinity of three different linear polyethylenimines (PEIs), namely LPEI, PEI“Max” and PEIpro™ that are commercially available. We have shown that PEI"Max" and PEIpro are more efficient than LPEI to transfect HEK293 and CHO cells [1]. Polydispersities and apparent sizes of LPEI, PEI“Max” and PEIpro™ were assessed by SEC (Fig. 1). Thanks to their net positive charges, PEIs are able to condense plasmid DNA (pDNA) and their respective pDNA affinity were compared (Fig. 2).

## 2 Experimental design, materials and methods

### 2.1 Size exclusion chromatography

Size exclusion chromatography (SEC) was performed on the liquid chromatograph GE ÄKTA explorer (Amersham Pharmacia) consisting from automated sample injector (loop, 0.5 mL), detector UV-900 (GE Healthcare Life Sciences), degasser of eluent and a chromatographic column (Superdex_200_10/300_GL from GE Healthcare Life Sciences, Cat.17-5175-01). SEC analysis was performed at room temperature using PBS as liquid phase. A volume of 0.5 mL of each PEI solution was injected and signals were recorded at a wavelength of 214 nm. Fractions of 0.5 mL were collected at a flow rate of 0.5 mL/min.

The size and polydispersity of a linear polyethylenimine (PEI) are critical parameters that influence buffering capacity, ability to condense plasmid DNA and cytotoxicity [3], [4], [5], [6]. According to manufacturer׳s product description, LPEI and PEI“Max” (Polyscience, Inc.) exhibit a molecular weight of 25 kDa (non-hydrochloride salt form). However, the size of PEIpro™ (Polyplus Transfection) is not disclosed by the manufacturer. Data presented here provide qualitative information about the apparent size of the three commercially available PEIs. Propionyl groups present on the polymer chain can be detected at 214 nm and the resulting UV chromatogram following size exclusion chromatography is shown in Fig. 1. Data indicated that LPEI and PEI“Max” exhibit the same size and polydispersity whereas PEIpro™ is significantly less polydisperse and elutes from the column with an higher apparent size. To allow the detection of fully deacylated polymer (absence of N-propionyl side chains) that would not absorb at 214 nm, an acid azo Orange II dye assay [7] was performed on each fraction and confirmed that the data showed in Fig. 1 are representative of the complete profile of each polyethylenimine. Moreover, as the intensity of the UV signals (integration of the surface under the curve) presented in Fig. 1 depends on the degree of acylation (presence of N-propionyl side chains), it also indicates that LPEI is the most acylated polymer whereas PEI“Max” and PEIpro™ are the least acylated.

### 2.2 Plasmid DNA affinity

For DNA gel retardation assay, increasing amounts of PEIs and 0.7 μg of pTT-SEAP DNA were each diluted in 10 μL final volume of F17 medium and vortexed. The diluted PEI was added to the DNA solution and the resulting mixture was gently vortexed. After 10 min incubation at room temperature (RT), the complex mixture was loaded onto a 1% (w/v) agarose gel. Electrophoresis was carried out in a Tris-acetate-EDTA (TAE) pH 7.4 running buffer solution. Visualization of DNA was performed using an Image Station 440 (Kodak Digital Science).

Agarose gel retardation electrophoresis (Fig. 2) was performed to evaluate the DNA condensation ability of the PEIs. The polyplexes were prepared with plasmid DNA at DNA:PEI ratios ranging from 0 to 0.5 (w:w) in 150 mM NaCl pH 7.4 and incubated for 10 min at RT. Polyplex suspensions containing 0.7 μg of DNA were electrophoresed on 1% agarose gels in 1× TAE running buffer pH 7.4 at 200 V for 20 min. The migration of complexed DNA on agarose gel was compared to that of naked pDNA. Data show that the migration of the DNA was progressively retarded with larger amounts of polymers, indicating an increasing complexation with PEI and progressive neutralization of negative phosphate charges. An increase in complex size might also explain the DNA retardation phenomenon. PEIpro™ almost completely complexed DNA with no observable streak at a DNA:PEI ratio of 1:0.4 (w:w) while this ratio was of 1:0.5 for LPEI and PEI“Max”. No DNA migration were observed at DNA:PEIs ratio superior to 1:0.5 (data not shown) meaning that DNA were fully complexed by all three PEIs.
